# Management Strategies for *Pasteurella multocida* Infections in Implant-Based Breast Reconstruction: Review of the Literature and a Successful Nonsurgical Salvage Case

**DOI:** 10.1093/asjof/ojag151

**Published:** 2026-07-16

**Authors:** Melike Refia Taslipinar, Yigit Yeni, Lal Sude Gucer, Billur Sezgin

## Abstract

*Pasteurella multocida* is a rare but clinically significant zoonotic pathogen in implant-based breast reconstruction, often related to pet exposure. Although implant infections typically lead to explantation, recent evidence—including our case—suggests that nonsurgical salvage may be possible in selected scenarios under close surveillance. In this report, the authors conducted a narrative review of the literature, identifying 5 previously reported *P. multocida* breast prosthesis infections and 1 *Pasteurella canis* case reviewed separately for broader zoonotic context. Among the 5 previously reported *P. multocida* cases, 4 required explantation, whereas only one—managed surgically—achieved successful expander salvage. This case report represents an additive contribution to this literature as the first reported instance of entirely nonsurgical expander salvage in the setting of *P. multocida* infection. Early microbiological diagnosis, appropriate antibiotic therapy, adjunctive bedside irrigation, and vigilant inpatient monitoring were key elements of this approach. This report highlights a safety-focused conservative management strategy, emphasizing that early-phase infections with low-virulence, beta-lactam-susceptible organisms in immunocompetent patients may not always require immediate explantation in the absence of clinical deterioration. Nonsurgical salvage deserves further investigation as a possible alternative to explantation in carefully selected cases.

**Level of Evidence**: 4 (Therapeutic) 
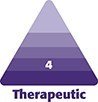

Periprosthetic infection is a significant complication following implant-based breast reconstruction, leading to prolonged hospitalization, additional surgical intervention, and ultimately implant loss.^1,2^ These infections typically arise through 2 main mechanisms: early infections are often linked to intraoperative contamination and hematoma, whereas late-onset infections are usually attributed to hematogenous seeding or external contamination through drains, suture tracts, or repeated percutaneous interventions, the majority of which are attributed to 5 microorganisms in the literature: *Staphylococcus aureus*, *Staphylococcus epidermidis*, *Pseudomonas aeruginosa*, *Propionibacterium*, and *Corynebacterium*.^3,4^ However, zoonotic organisms such as *P. multocida*—a gram-negative coccobacillus residing in the oral cavities of domestic animals—are rare but relevant pathogens.^5-9^ Transmission typically follows animal bites, scratches, or saliva contact with open wounds or surgical drains.^5-9^ The presence of surgical drains is a known risk factor for implant infection, particularly when contaminated following discharge, highlighting the need for strict postoperative wound care protocols.^1^ In this article, we present a successfully salvaged case using nonsurgical therapy and then review the existing literature on *P. multocida* breast implant infections to contextualize our findings and provide a framework for early recognition, safety-focused decision making, and the limited circumstances under which conservative treatment was successful in this patient.

## CASE PRESENTATION

**Figure 1.**
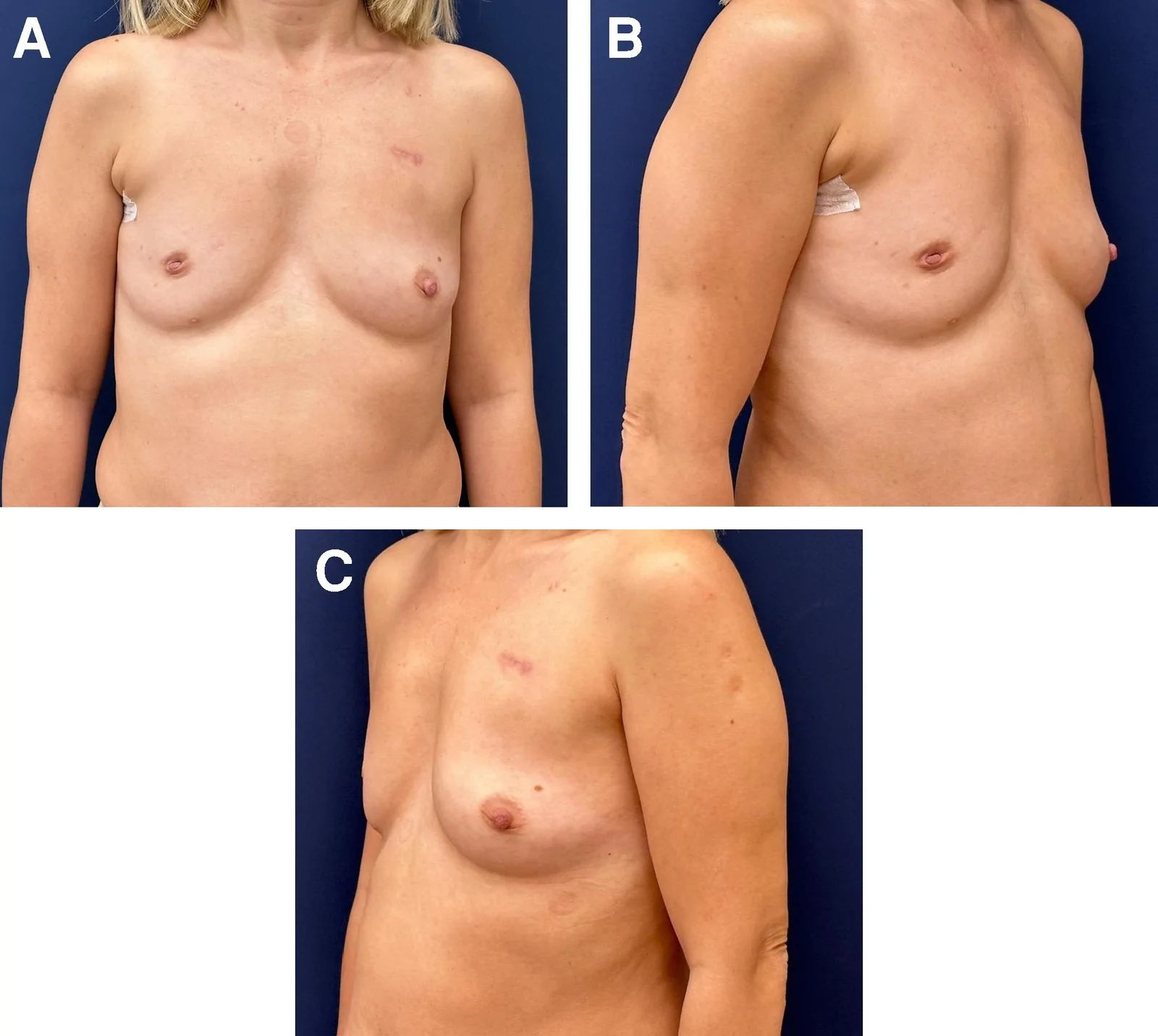
(A) A 42-year-old female patient with right-sided breast cancer, preoperative frontal view, (B) oblique view, right, and (C) oblique view, left.

A 42-year-old female patient with a history of invasive ductal carcinoma (Figure 1A-C) of the right breast underwent a right-sided therapeutic skin-sparing mastectomy with sentinel lymph node biopsy along with a left-sided prophylactic skin-sparing mastectomy and bilateral prepectoral breast reconstruction using Motiva Flora 350 cc (Establishment Labs Holdings Inc., Alajuela, Costa Rica) tissue expanders without acellular dermal matrix (ADM) following neoadjuvant chemotherapy. Perioperative antibiotic prophylaxis was administered with a first-generation cephalosporin (cefazolin 2 g intravenous [IV]) per institutional protocol. As part of our routine practice for all implant-based breast reconstruction procedures, intraoperative pocket irrigation was performed bilaterally with 1000 cc of normal saline solution containing 1 vial each of 250 mg/3 mL rifamycin and 160 mg/2 mL gentamicin before expander insertion. Right and left expanders were inflated intraoperatively with 120 and 150 cc sterile saline, respectively. Two Jackson–Pratt drains were placed on the right side: 1 in the breast pocket and 1 in the axilla. One drain was placed in the left breast pocket. The postoperative course was initially uneventful, and the patient was discharged 2 days postoperatively. The axillary drain on the right side had been removed before discharge; the remaining right breast pocket drain and the left breast pocket drain were retained in situ at the time of discharge.

**Figure 2.**
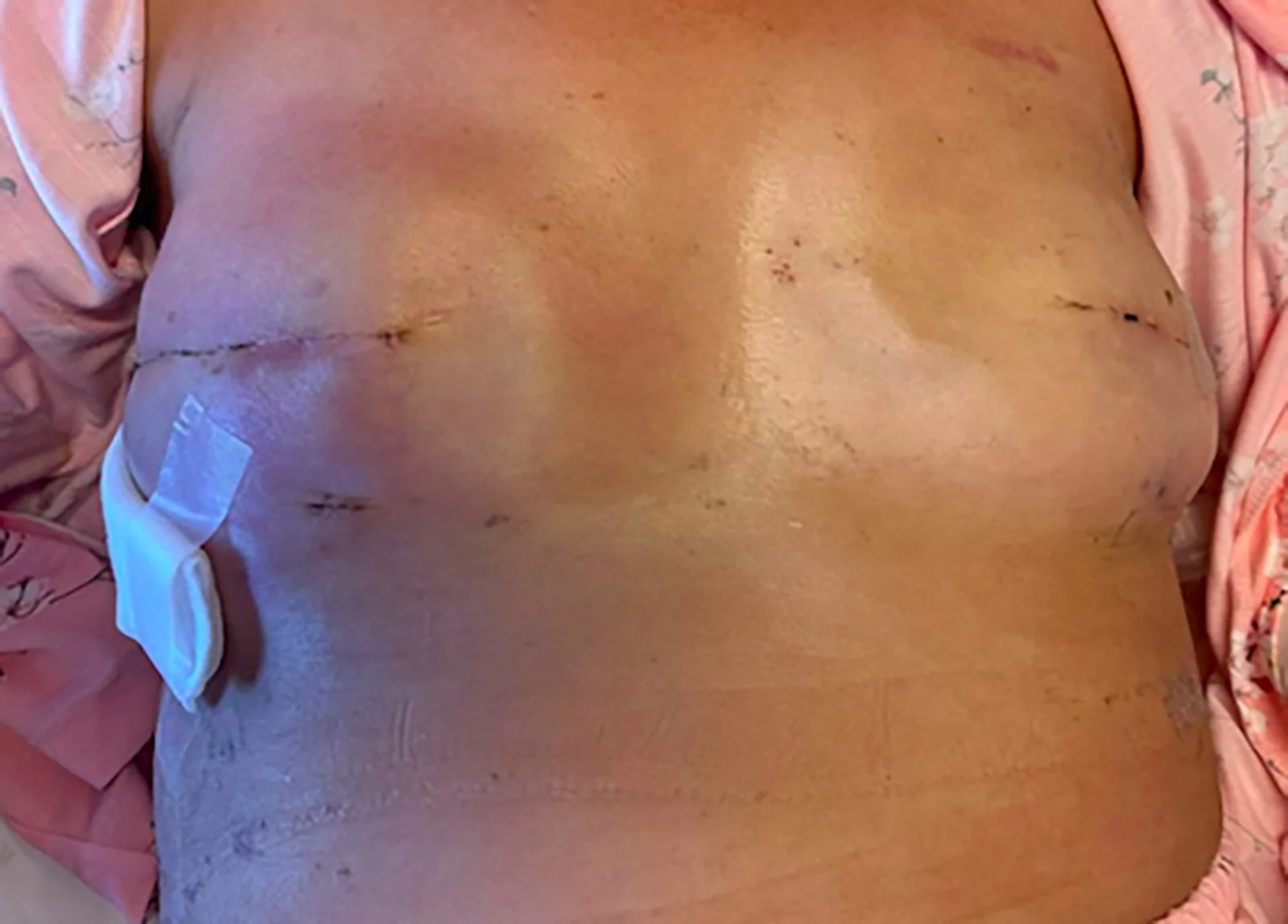
Bedside image of breasts at the day of admission, postoperative Day 12. Note the erythema around right breast. Image quality is limited because of acute bedside setting; standardized clinical photography was not feasible during the inpatient admission.

On postoperative Day 12, the patient presented with fever and chills accompanied by erythema, edema, and pain in the right breast (Figure 2). Drain discharge in the right breast pocket drain tube appeared purulent with a cottage cheese-like consistency. The right breast pocket drain was immediately removed and the area was gently massaged to evacuate the remaining collection. A sterile specimen of the drainage fluid was collected immediately and submitted for microbiological culture. Laboratory findings revealed leukocytosis (26 × 10^9^/L; normal: 4-10 × 10^9^/L) and an elevated C-reactive protein level (66.7 mg/L; normal: <5 mg/L). Upon further questioning, the patient reported that her cat had chewed on the right drain tubing 2 days before while she was asleep.

The patient was immediately admitted for inpatient treatment and active surveillance. Empirical intravenous antibiotic therapy was initiated with piperacillin–tazobactam (4.5 g IV every 6 h). Concurrently, bedside irrigation of the periprosthetic pocket was performed by the attending plastic surgeon. With the patient in the left lateral decubitus position, a blunt-tipped 20 cc syringe without the needle end was inserted directly into the drain tract incision. The drain tract was kept open (not sutured) throughout the treatment period. Successful introduction of irrigant into the pocket was confirmed by monitoring for fluid egress from the wound and the absence of significant resistance and by the patient's tolerance of the procedure. The wound was dressed with sterile gauze between sessions. For the first 2 days, the periprosthetic pocket was irrigated twice daily, first with 1000 cc sterile saline and then with rifampicin (600 mg in 100 cc saline). By Day 3, cultures confirmed *P. multocida* infection. In consultation with the infectious diseases service, piperacillin–tazobactam was continued for a total of 7 days, given the severity of the initial presentation with systemic signs (fever, leukocytosis, and elevated inflammatory markers), without de-escalation to an intermediate parenteral agent. Irrigation was continued for 5 additional days twice daily with 1000 cc sterile saline followed by an ampicillin–sulbactam solution (1000 mg ampicillin/500 mg sulbactam in 100 cc saline). Symptoms resolved and inflammatory markers normalized. Upon completion of the intravenous course, the patient was discharged directly on a 2-week course of oral amoxicillin–clavulanate (1000 mg/125 mg, 3 times daily). Repeat cultures were sterile. The patient remained afebrile with complete resolution of inflammation and no subsequent need for expander removal.

**Figure 3.**
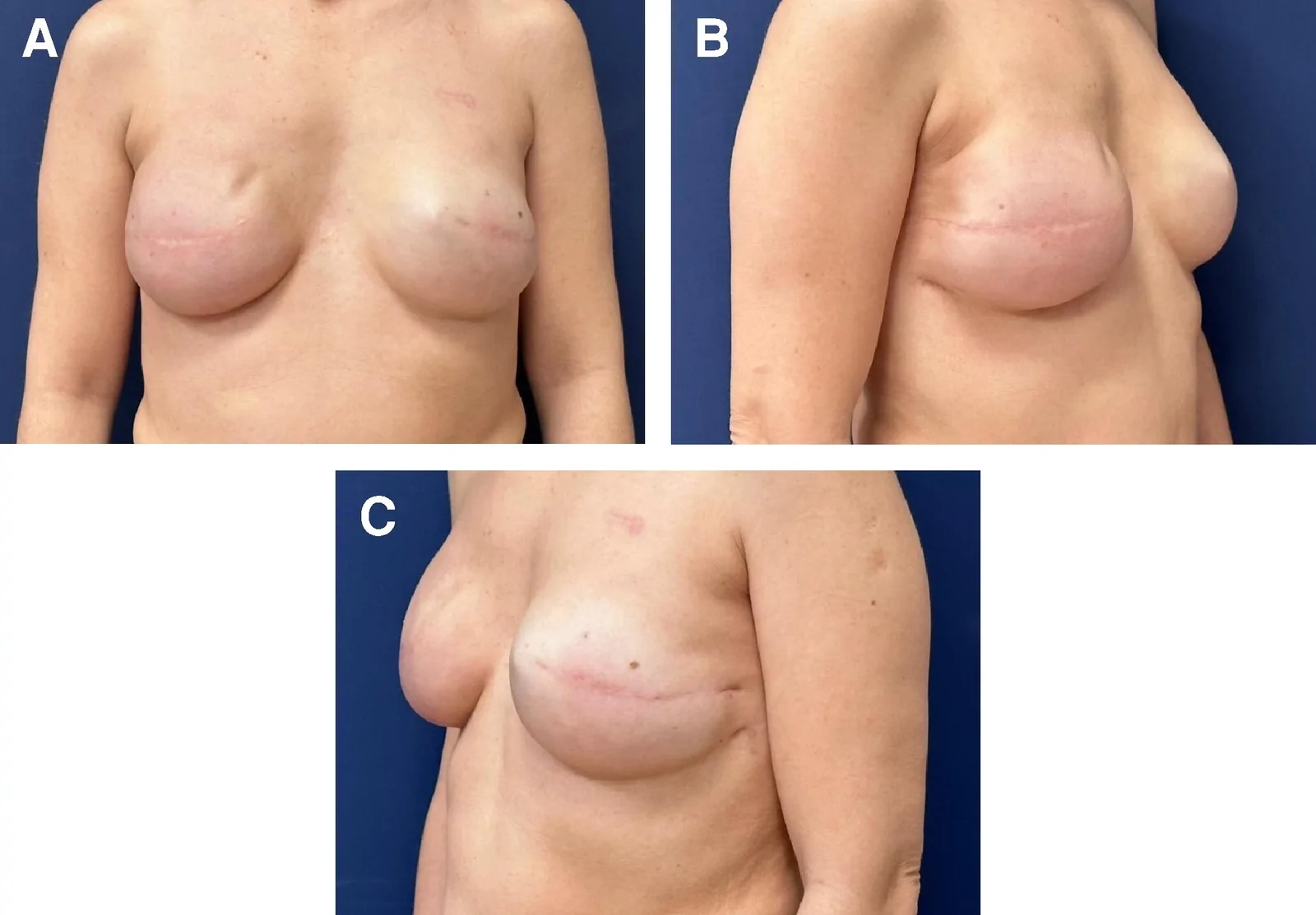
(A) Postoperative third month following bilateral skin-sparing mastectomy with expander-based breast reconstruction, upon completion of adjuvant radiation treatment, frontal view, (B) oblique view, right, and (C) oblique view, left.

**Figure 4.**
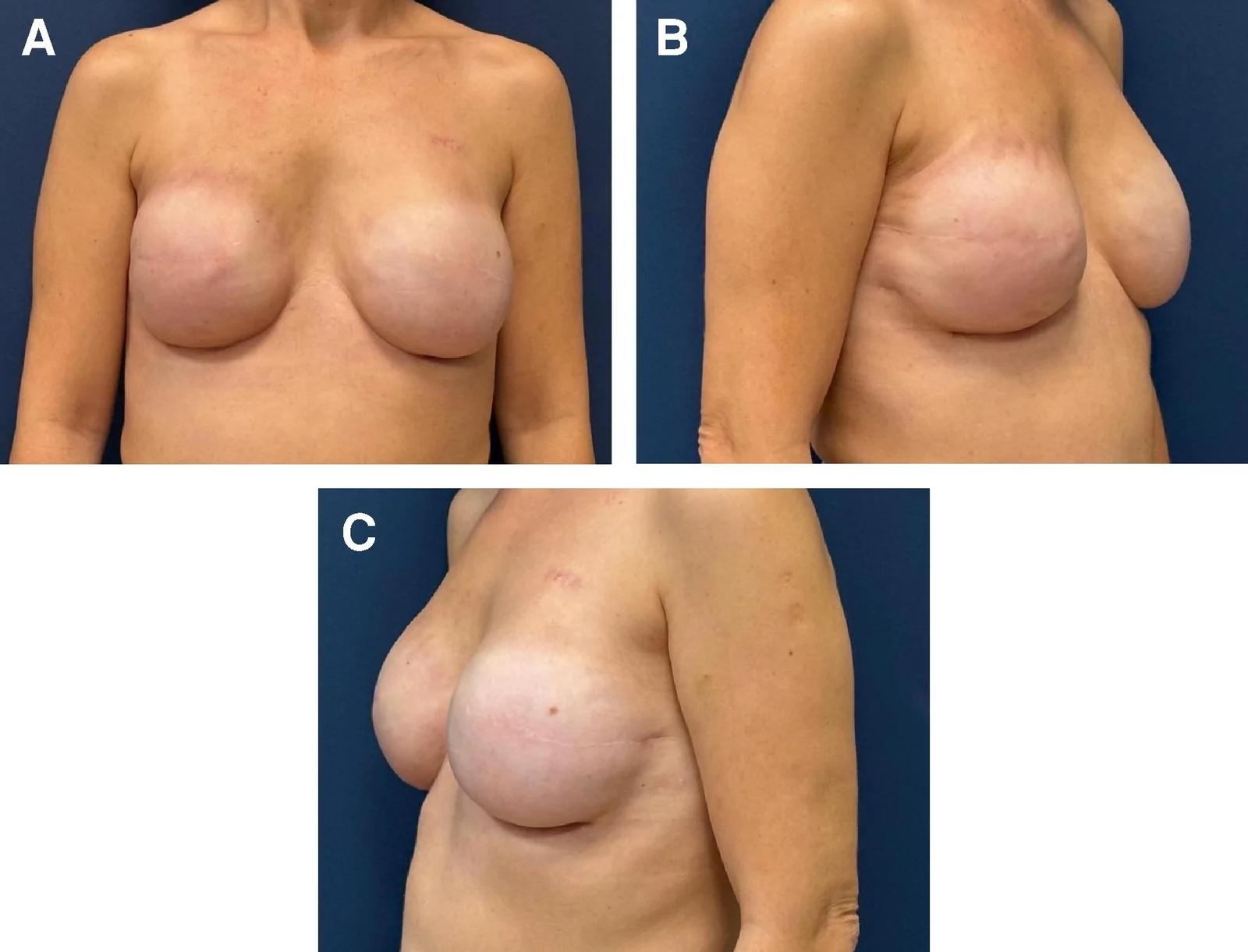
(A) Postoperative third month following second stage breast reconstruction with silicone implants and fat grafting, obtained ∼1 year after the initial mastectomy and tissue expander reconstruction, ∼11.5 months after the *Pasteurella multocida* infection, frontal view, (B) oblique view, right, and (C) oblique view, left.

After recovery, the patient successfully completed adjuvant radiotherapy without complications (Figure 3A-C). Ten months following the initial procedure, the tissue expanders were electively exchanged for silicone breast implants (Motiva Ergonomix 2 SmoothSilk demi, 405 cc; Establishment Labs Holdings Inc.) with autologous fat grafting (∼80 cc to the right breast and 60 cc to the left breast, targeting the upper pole and mastectomy flap interfaces) to improve contouring. No macroscopic signs of infection-related sequelae were noted in the right implant pocket or capsule. Nevertheless, meticulous intraoperative precautions were taken, including the use of separate surgical instruments and gloves for each breast to prevent potential cross-contamination. The postoperative course was uneventful, with no infection-related or other complications, and the patient reported a satisfactory aesthetic outcome ∼1 year after the initial mastectomy and tissue expander reconstruction, ∼11.5 months after the *P. multocida* infection and postoperative third month after the implant exchange and fat grafting (Figure 4A-C).

## LITERATURE REVIEW

A narrative review of the literature was conducted to identify published articles on *Pasteurella* infection of breast prosthetics. Google Scholar and PubMed databases were searched using the following keywords and Boolean operators: (“Pasteurella multocida” OR “Pasteurella canis”) AND (“breast implant” OR “tissue expander” OR “breast prosthesis”). The search was not limited by date of publication. To ensure that no relevant cases were missed, the search terms also included “zoonotic infection” and “cat bite” in combination with “breast reconstruction.” After identifying initial articles, the snowballing method was used by reviewing the reference lists of related articles to identify further relevant publications.

Five cases of *P. multocida* infection in breast prostheses were identified in the literature.^5-9^ Additionally, one case of *Pasteurella canis* was found and reviewed to further analyze the risk of zoonotic infections from *Pasteurella* species.^10^ Data focusing on mechanism of injury, type of surgical intervention, management, and outcome are summarized in Table 1.

**Table 1.** Overview of *Pasteurella multocida*–Associated Breast Implant Infections Reported in the Literature

Author (year)	Patient age	Animal contact	Surgery type	Management	Outcome
Johnson et al (2000)^5^	44	Close contact with domestic cats	Tissue expander	IV Abx, temporary improvement	Reinfection, explantation
Mathieu et al (2008)^6^	46	Close contact with domestic dogs	Tissue expander	IV Abx+ surgical debridement	Explantation
Martinez et al (2014)^7^	58	Cat bit the drain tubing	Tissue expander + ADM	IV Abx + surgical debridement + triple antibiotic irrigation	Expander salvaged
Ruffenach et al (2021)^9^	40	Cat bit the drain tubing	Tissue expander	IV Abx + surgical debridement	Explantation
Lenne et al (2016)^8^	33	Cat scratch near the drain	Silicone implant + decellularized facia lata	IV Abx + surgical debridement	Explantation

ADM, acellular dermal matrix; IV Abx, intravenous antibiotic therapy.

Johnson et al reported the first such case, initially managed conservatively, but explantation was ultimately required because of recurrence.^5^ Mathieu et al and Ruffenach et al similarly described implant infections following close pet exposure that necessitated surgical removal of the prosthesis.^6,9^ Lenne et al reported a case involving drain area trauma from a cat scratch that also led to explantation.^8^ Martinez et al remains the only previously published case where the expander was salvaged—through immediate surgical debridement and triple antibiotic irrigation.^7^ A summary of all reported *P. multocida* breast implant infections in the literature is provided in Table 1.

## DISCUSSION

*Pasteurella multocida* is a small, nonmotile, facultative anaerobic gram-negative coccobacillus which often colonizes the oropharynx of cats and dogs.^11,12^ It is most commonly detected as a skin and soft tissue infection following animal trauma.^13^ Although rarely implicated in breast implant infections, when it occurs, symptom onset can be rapid—within hours of inoculation.^7^ Transmission is most often associated with animal bites or scratches, but other routes such as saliva contamination or direct pet contact with surgical drains have also been reported.^5-9^

The present case adds to a very limited body of literature documenting *P. multocida* infection of breast prostheses. It is important to emphasize that the broader literature on tissue expander infections demonstrates a poor prognosis when systemic signs are present. Overall salvage rates across all infection grades range from ∼58% to 75% in large series; however, these figures deteriorate substantially when deep pocket infection is accompanied by fever and leukocytosis.^1,2^ Viola et al specifically demonstrated that patients requiring explantation had significantly higher temperatures at presentation and were significantly more likely to have deep-seated rather than superficial infections, with salvage rates dropping to 0% for *Pseudomonas* and rising to 92% only in culture-negative cases.^14^ Furthermore, organism virulence and biofilm capacity are critical determinants: in one series of 300 expanders, 57% of infected cases ultimately required explantation after failed conservative treatment.^15^ Our patient presented with marked leukocytosis (26 × 10^9^/L), fever, and purulent drainage—a clinical profile that, based on the available literature, would be expected to carry a high risk of explantation. Our outcome must therefore be considered potentially exceptional rather than a predictable response to conservative management and should not be interpreted as evidence that expander retention is broadly safe or effective in similar presentations. Clinicians must exercise careful judgment, maintain a low threshold for surgical intervention, and ensure close inpatient monitoring if a conservative approach is attempted.

The favorable outcome in this patient may have been related to the interaction of organism biology, timing, and intensity of local and systemic therapy. *Pasteurella multocida* differs from many common implant-associated pathogens in several biologically relevant ways. Unlike *S. epidermidis* or *Ps. aeruginosa*—which are highly adept at biofilm formation and often render implant infections refractory to antibiotics—*P. multocida* demonstrates significantly less robust biofilm-forming capacity in vitro, and its clinical biofilm relevance remains less established.^16-19^ Furthermore, *P. multocida* is generally susceptible to beta-lactam antibiotics, enabling reliable targeted therapy once cultures are available. These characteristics may offer a narrow therapeutic window for conservative intervention before a stable biofilm matrix develops. In our case, bedside irrigation was started within 48 h of symptom onset and was paired with systemic beta-lactam therapy and local antibiotic irrigation. Biofilms protect bacterial colonies from immune surveillance and drug penetration, making eradication difficult once mature.^16,17^ The early timing of irrigation, the organism's susceptibility profile, and the absence of clinical deterioration likely contributed to expander retention. In contrast, infections caused by more biofilm-competent or resistant organisms, inadequate antibiotic regimens, insufficient irrigation, or delayed clinical response would be expected to have a substantially higher risk of treatment failure and should prompt operative source control rather than continued observation. These practices are consistent with principles described in best-practice protocols for implant salvage in biofilm-susceptible environments.^20,21^ We also note that our routine use of intraoperative pocket irrigation with an antibiotic solution (rifampicin and gentamicin in normal saline) may have contributed to a lower initial bacterial burden, potentially preserving a window for conservative management had contamination occurred. Although not specific to this pathogen, intraoperative antimicrobial irrigation is a recognized strategy to reduce biofilm-associated infection risk in implant-based surgery.^16^

An additional clinical challenge inherent to this approach is that culture results typically require 48 to 72 h to finalize. In our case, empirical therapy was initiated promptly and the organism proved susceptible. However, a clinician committing to a conservative approach must acknowledge that if the responsible organism is less susceptible or more virulent, the delay in definitive surgical management could worsen the patient's outcome. This underscores the importance of close inpatient surveillance, serial clinical assessment, inflammatory marker monitoring, infectious diseases collaboration, and a predefined threshold for transition to operative management. In our case, the decision to continue piperacillin–tazobactam for the full 7-day inpatient course—rather than de-escalating upon culture confirmation—reflected the severity of the initial presentation with systemic features and was made in close collaboration with the infectious diseases service.

Emerging evidence suggests that implant surface characteristics may also play a role in bacterial adhesion and biofilm formation. Surface topography has been shown to influence microbial attachment, with smooth or microtextured surfaces potentially demonstrating reduced bacterial colonization compared with macrotextured implants.^16,17,22^ Experimental and clinical studies indicate that surface architecture may affect the initial stages of biofilm development, which in turn could influence the likelihood of successful implant salvage. Although direct clinical evidence comparing salvage outcomes across implant surface types remains limited, it is conceivable that implants with reduced bacterial adherence profiles may offer a more favorable environment for conservative management in early-phase infections. Further investigation into the relationship between implant surface properties and infection outcomes may help refine patient selection and optimize salvage strategies.

Additionally, emerging evidence links subclinical biofilm infection to capsular contracture, chronic inflammation, and poor reconstructive outcomes—even in the absence of overt infection.^23^ The absence of macroscopic sequelae in our patient supports the possibility that early and aggressive treatment may eradicate infection without implant loss in selected circumstances; however, this should not be generalized to patients with persistent systemic signs, inadequate source control, resistant organisms, or delayed response.

The decision to attempt conservative salvage in this case was based on several converging favorable factors: early presentation within the likely prebiofilm window (postoperative day 12); identification of a low-biofilm-forming, consistently beta-lactam-susceptible organism; the patient's immunocompetent status; a clearly identified mechanism of contamination enabling targeted therapy; and a rapid initial clinical response to irrigation and empirical antibiotics. These factors together created a narrow but potentially favorable window in this individual patient. It should be emphasized, however, that the approach was not passive observation: it required inpatient admission, active bedside source reduction through repeated irrigation, broad-spectrum intravenous therapy followed by targeted oral therapy, and close collaboration with infectious diseases specialists. Surgical management—including operative washout, expander exchange, or explantation—remained immediately available and would have been pursued at the first sign of clinical deterioration or inadequate response.

The risks of delayed or inadequate management of infected tissue expanders must not be underestimated. Failure to achieve adequate source control carries the risk of progressive deep periprosthetic infection, bacteremia, sepsis, prolonged hospitalization, delays in oncologic treatment, reconstructive failure, and additional morbidity. In a patient with worsening systemic status, persistent fever or leukocytosis, spreading cellulitis, hemodynamic instability, or failure of inflammatory markers to improve, retention of an infected expander may convert a potentially controllable infection into a severe systemic illness. The conservative approach described here was pursued only in the context of active daily inpatient monitoring, infectious diseases consultation, and readiness for immediate operative intervention. It should not be interpreted as a strategy to defer necessary surgery.

The use of ADM in infected breast reconstructions remains controversial. In the only previously reported case of successful expander salvage—Martinez et al—ADM was used and yet implant retention was achieved through immediate surgical debridement and triple antibiotic irrigation.^7^ This suggests that ADM does not preclude salvage when aggressive management is employed early.^7^ Conversely, other reported cases without ADM resulted in explantation despite antibiotic treatment, further highlighting the role of timing, virulence, and host factors rather than ADM presence alone.^5,6,8,9^ In our case, ADM was not utilized, which may have contributed to more efficient antibiotic penetration and reduced microbial adherence to surrounding tissue. However, given the heterogeneity of reported cases, this observation cannot be generalized. If ADM had been present in our patient, a lower threshold for surgical debridement might have been warranted, given theoretical concerns about limited antibiotic diffusion through the matrix and its potential as a scaffold for bacterial persistence. Further studies are warranted to determine whether ADM presence influences the feasibility and success of conservative management in this setting.

Further support for individualized decision making is found in the prosthetic joint infection (PJI) literature, where *P. multocida* has been more frequently encountered. Honnorat et al reported a series of PJI cases where some prostheses were successfully retained using antibiotics alone, whereas others required removal—highlighting the variability in clinical course and response to conservative therapy.^18^ A case reported by Hannouille et al involving *P. canis* in breast implant infection further broadens the spectrum of zoonotic threats in breast reconstruction and reinforces the relevance of proper pet-related postoperative hygiene and surveillance.^10^

Infections involving surgical drains represent a well-known but sometimes underemphasized risk in implant-based surgery. Although drains help reduce seroma formation, they may inadvertently serve as entry points for pathogens—particularly when left in place postdischarge.^1^ In our case, the drain tubing, once punctured by a household cat, likely allowed atmospheric exposure to the previously sealed periprosthetic space. This sudden loss of negative pressure may have facilitated a rapid influx of oral flora into the surgical pocket. Rozen et al recently documented a case of *P. multocida* infection following abdominoplasty through a drain that had been punctured and then sealed with household adhesive, further underscoring the vulnerability of drainage systems to zoonotic contamination when the negative pressure environment is compromised.^24^ Therefore, although drains remain indispensable in many reconstructive procedures, their management after discharge—especially in pet-owning patients—must be accompanied by strict hygiene instructions and awareness of infection risk.

This review underscores not only the importance of considering zoonotic pathogens in implant infections but also the possibility that nonsurgical salvage may be feasible in a narrow subset of cases characterized by early diagnosis, a low-virulence and antibiotic-susceptible organism, an immunocompetent host, effective local irrigation, and rapid clinical improvement. Education on pet-related wound contamination, proper drain care, and postdischarge precautions should be emphasized for all patients undergoing implant-based breast reconstruction. Equally important, attempts at retention must be governed by patient safety, with prompt conversion to operative source control when clinical response is incomplete or deterioration occurs.

### Limitations

This report has several important limitations that must be acknowledged. First, as a single case report, no generalizable conclusions can be drawn, and the outcome should be considered potentially exceptional rather than representative. Second, the conservative approach described may not be safe or effective for organisms with greater biofilm-forming capacity or antibiotic resistance profiles; clinicians must await culture results with caution and be prepared to escalate to surgical management promptly. Third, the technique of bedside pocket irrigation through the drain tract incision is operator dependent and may not be feasible in patients with a larger body habitus, thicker subcutaneous breast tissue, or where the drain tract has closed. Fourth, the admission photograph (Figure 2) is of limited quality because of the acute bedside setting, and additional inpatient photographs documenting the clinical course were not available. Fifth, the risk of worsening infection exists if irrigation is inadequate, drainage is incomplete, antibiotic selection is not appropriately targeted, or operative source control is delayed despite clinical deterioration. These limitations collectively underscore that clinical judgment and individualized patient assessment are essential and that this case and approach may not be applicable in all infections or clinical settings.

## CONCLUSIONS

*Pasteurella multocida*, while rare, represents a significant zoonotic risk in implant-based breast reconstruction, particularly in patients living in households with pets. This case reinforces the importance of strict drain care protocols and early microbiological assessment in the event of postoperative inflammation. It suggests that nonsurgical management of breast implant infections may be possible in carefully selected patients with early-phase infections caused by low-virulence, antibiotic-susceptible organisms when effective antibiotic therapy, repeated irrigation, close inpatient monitoring, and rapid clinical improvement are present. However, the successful outcome in this patient should be considered exceptional rather than typical, and expander retention must not delay operative source control in patients with worsening infection, persistent systemic signs, resistant organisms, or inadequate response to therapy. These findings must be interpreted with caution given the single-case nature of this report, the absence of previous data on nonsurgical management of *Pasteurella* expander infections, and the generally poor salvage rates reported in the literature for deeply infected tissue expanders with systemic involvement. Further data are needed before this approach can be broadly recommended.
